# Evaluating dispersal potential of an invasive fish by the use of aerobic scope and osmoregulation capacity

**DOI:** 10.1371/journal.pone.0176038

**Published:** 2017-04-19

**Authors:** Jane W. Behrens, Mikael van Deurs, Emil A. F. Christensen

**Affiliations:** 1 National Institute of Aquatic Resources, Technical University of Denmark, Charlottenlund, Denmark; 2 Marine Biological Section, University of Copenhagen, Helsingør, Denmark; University of Tasmania, AUSTRALIA

## Abstract

Non-indigenous species (NIS) can impact marine biodiversity and ecosystem structure and function. Once introduced into a new region, secondary dispersal is limited by the physiology of the organism in relation to the ambient environment and by complex interactions between a suite of ecological factors such as presence of predators, competitors, and parasites. Early prediction of dispersal potential and future ‘area of impact’ is challenging, but also a great asset in taking appropriate management actions. Aerobic scope (AS) in fish has been linked to various fitness-related parameters, and may be valuable in determining dispersal potential of aquatic invasive species in novel environments. Round goby, *Neogobius melanostomus*, one of the most wide-ranging invasive fish species in Europe and North America, currently thrives in brackish and fresh water, but its ability to survive in high salinity waters is unknown to date. We show that AS in round goby is reduced by 30% and blood plasma osmolality increased (indicating reduced capacity for osmoregulation) at salinities approaching oceanic conditions, following slow ramping (5 PSU per week) and subsequent long-term acclimation to salinities ranging between 0 and 30 PSU (8 days at final treatment salinities before blood plasma osmolality measurements, 12–20 additional days before respirometry). Survival was also reduced at the highest salinities yet a significant proportion (61%) of the fish survived at 30 PSU. Reduced physiological performance at the highest salinities may affect growth and competitive ability under oceanic conditions, but to what extent reduced AS and osmoregulatory capacity will slow the current 30 km year^-1^ rate of advance of the species through the steep salinity gradient from the brackish Baltic Sea and into the oceanic North Sea remains speculative. An unintended natural experiment is in progress to test whether the rate of advance slows down. At the current rate of advance the species will reach the oceanic North Sea by 2018/2019, therefore time for taking preventative action is short.

## Introduction

Non-indigenous species (NIS) can have strong impacts on marine biodiversity and ecosystem structure and functions [1–3]. Hence, disentangling invasion mechanisms is of utmost ecological, economic, and social importance. In some European Seas, the number of NIS have grown by more than 200% within the last 45 years, with shipping activities emerging as the primary vector of introductions [4]. While control of ballast water may aid to minimize the risk of transfer to yet uncolonized areas, post-invasion management actions have lately received substantial attention [5–10]. Effective post-invasion actions are hindered by the difficulty of predicting the direction and rate of secondary dispersal (i.e. by natural migration or dispersal) following introduction into a new region.

The most fundamental constraints on distribution of a species arise from physiological limitations and performance in response to the ambient environment [11–15]. For a NIS in a new region, secondary dispersal also depends on complex interactions between a suite of ecological factors, such as presence of predators, competitors, and parasites [16–18]. The key traits defining the physiological performance of a NIS must be identified, together with the responses of these traits to ambient environmental variation, if we are to accurately predict the ultimate distribution and potential impacts that may result from an invasion [19].

Fry [20] introduced the idea that any form of exercise and growth are fueled by the difference between a fish’s maximal and minimal rates of metabolism, termed the scope for activity. Fry also explained that environmental factors like temperature, water chemistry and gas concentration affect scope for activity, and thus the general performance of the individual. Aerobic metabolism, that is the maximum metabolic rate (MMR) [21] and standard metabolic rate (SMR)[22], has recently been used as an approximation of total metabolism [23]. Consequently, scope for activity is now used interchangeably with aerobic scope (AS = MMR—SMR) [24]. For the most part, AS has been used to evaluate the performance of individuals across temperature gradients, providing thermal windows of aerobic performance. AS is highest at the species' optimal temperature, and declines as towards the upper and lower thermal limits [25, 26]. Fish need stable internal ion content and composition to maintain homeostasis, which entails an energetic cost of osmoregulation (i.e. increased SMR) that may be substantial at salinities where large concentration gradients between the internal and external environment exist [27, 28]. Although fish can decrease gill permeability to decrease the osmotic water potential, this compromises gas exchange, often seen as reduced MMR (the osmorespiratory compromise [29, 30]). If SMR and/or MMR are affected by salinity stress, there will be a salinity window of tolerance beyond which AS is not sufficient to maintain osmotic homeostasis [31]. Consequently, blood plasma osmolality will deviate from the optimal, and the fish may eventually die [32].

Of Ponto-Caspian origin, round goby, *Neogobius melanostomus* (Pallas 1814), is today a widespread and very invasive NIS. Following its initial discovery in 1990 in the Gulf of Gdansk (Poland) and the St Clair River (US), it has now established large populations throughout the Baltic Sea, several major European inlet waters, and the North American Great Lakes [33–36]. From these established areas they continuously disperse actively [37–39]. Round goby prey on the eggs of native and commercially valuable fish and are often outcompete them for spawning habitats, shelter and food [40–46]. A recent evaluation of 18 taxa of NIS in the Baltic Sea region found round goby amongst those with the greatest impact [8].

**Fig 1:**
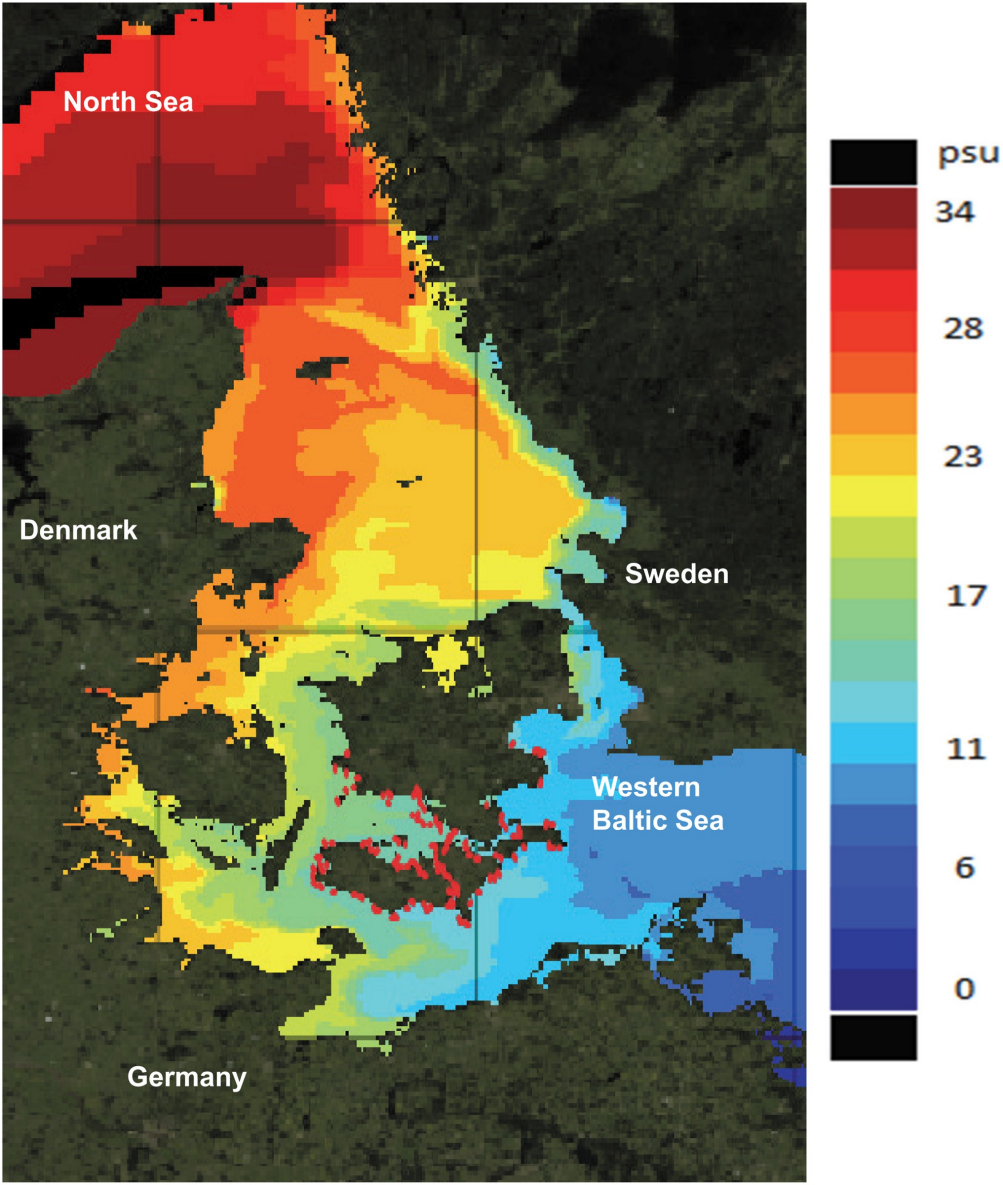
Occurrence of round goby (red dots, from Azour et al. [48]) in the Baltic Sea-North Sea transition zone in relation to salinity at 0-5m depth. Salinity data (yearly mean) are provided by Myocean and are output from the Baltic Sea 3D physical model code HBM (HIROMB-BOOS) with a spatial resolution of 2 km and temporal resolution of 1 hour.

The native ranges of round goby are large inland brackish water bodies (mean salinities of 12 PSU [47]). Invasion of habitats dissimilar to the native range is not uncommon and round goby has proved successful in invading not only brackish waters but also fresh water systems, however, no fully marine populations are known at present. We therefor hypothesize that the ability to maintain high physiological performance in a wide range of salinities is an important factor underpinning the success of this particular NIS. Indeed, round goby is currently expanding into areas of still higher salinities; from the most western part of the Baltic Sea secondary dispersal is taking place northward along the coastline at a rate of approximate 30 km year^-1^ [48]. This area comprises a transition zone between the brackish Baltic Sea and the full oceanic North Sea, where at present the range of the round goby covers salinities from approximately 10 to 17 PSU (Fig 1). An unintended natural experiment is thus in progress to test whether the rate of advance slows down.

By investigating the effect of salinity (0–30 PSU) on two physiological traits, namely AS and osmoregulation, the present study provides a mechanistic assessment of the secondary dispersal potential of the species within the transition zone between the brackish Baltic Sea and the oceanic North Sea, which is characterised by a steep salinity gradient. We expected that the highest AS for round goby would occur at near isoosmotic salinities (10PSU, the salinity at the location of origin of the experimental fish), with decreasing AS at salinities deviating from these values. We also expected that round goby would have limited ability to osmoregulate, that is, to maintain stable blood plasma osmolality, at higher salinities.

## Results

### Water quality

Initial [NH_4_^+^], [NO_2_^-^] and pH were high (up to 1 mg L^-1^ and around pH 8.4) in all tanks as the biota in the trickle filters were still settling. However, [NH_4_^+^], [NO_2_^-^] were on average 0.05 and 0.1 mg L^-1^, respectively, from at least two week before initiation of experiments and throughout the experimental period, and pH had stabilized at 7.9.

### Oxygen consumption rate

**Fig 2:**
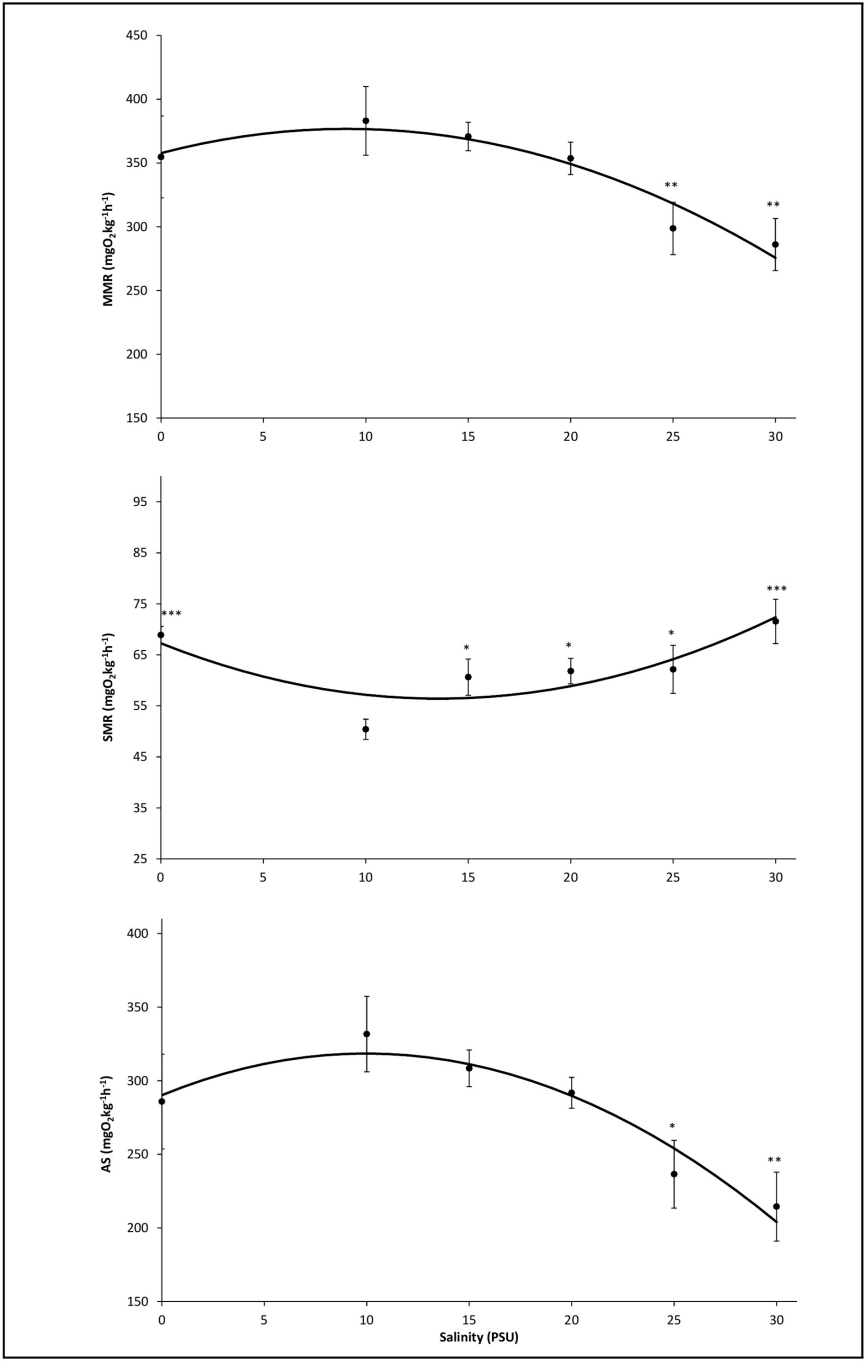
Relationship between ambient salinity and measured averages (circles, mean ± SE) and modelled (lines) round goby maximum metabolic rate (MMR, upper graph), standard metabolic rate (SMR, middle graph), and aerobic scope (AS: MMR minus SMR, lower graph). For all groups n = 8, except for 10 and 15 PSU where n = 7 for MMR and AS. * indicate p<0.05, ** indicate p<0.01 and ***indicate p<0.001.

Salinity affected all three response variables significantly, i.e. SMR, MMR, and AS (for SMR, one-way ANOVA F(5,42) = 4.908, p<0.01; for MMR, one-way ANOVA F(5,40) = 3.100, p = 0.02; for AS, one-way ANOVA F(5,40) = 3.642, p<0.01). The ANOVA model showed that MMR and AS were significantly lower in treatments 25 and 30 PSU, compared to the reference treatment (10 PSU) (p = 0.010 and 0.006, respectively). For SMR, all treatments were significantly higher than the reference treatment (10 PSU) (between p = 0.036 and p<0.001) (Fig 2).

In the parameterization, a second order term was significant for SMR (F = 14.883, P<0.001), MMR (F = 5.842, P = 0.02), and AS (F = 5.842, P = 0.006), and the second order polynomial regressions against salinity were significant for SMR (F(2,45) = 8.045, p < 0.001) with an R^2^ of 0.263, for MMR (F(2,43) = 7.49, p = 0.020) with an R^2^ of 0.258, and for AS (F(2,43) = 8.898, p < 0.006) with an R^2^ of 0.293 (Fig 2).

The equations were as follows:

$$MMR=4.194*SAL-0.231*SAL^{2}+357.665$$ (1)

$$SMR=-1.600*SAL+0.059*SAL^{2}+67.252$$ (2)

$$AS=5.683*SAL-0.285*SAL^{2}+290.127$$ (3)

where SAL is salinity.

### Survival

Survival during the 3 month experimental period was 95% at 10 and 15 PSU, but fell to 89%, 72% and 61% at 20, 25 and 30 PSU, respectively. In freshwater survival was as in 20 PSU (i.e. 89%).

### Blood plasma osmolality

**Fig 3:**
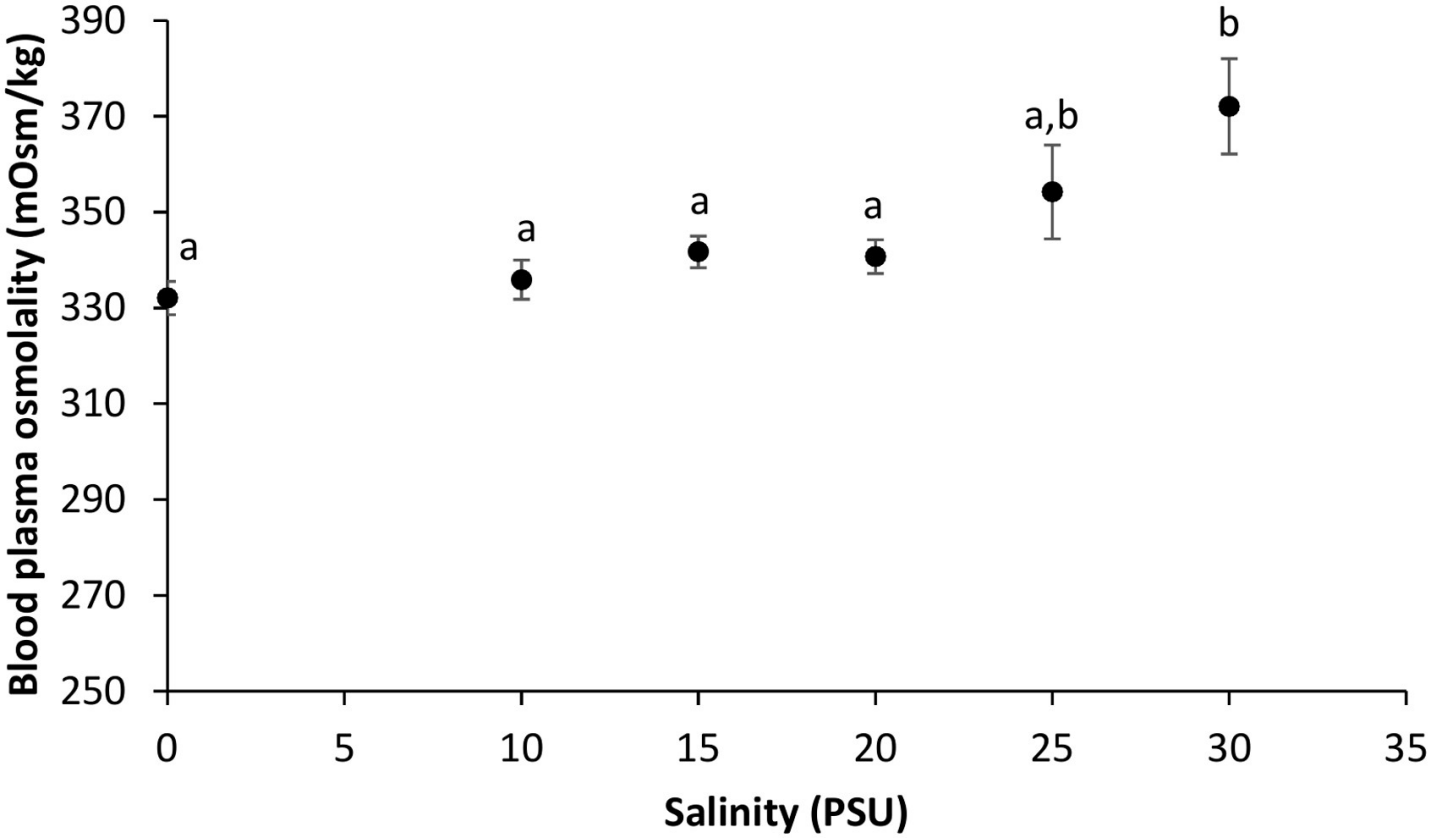
Relationship between ambient salinity and round goby plasma osmolality (mean ± SE). For all groups n = 10, except at 10 and 30 PSU where n = 9. Different letters denote significant differences in plasma osmolality between treatment groups.

There was a significant effect of salinity on blood plasma osmolality (Kruskal-Wallis test, p = 0.001). The blood plasma osmolality was not statistically different at salinities between 0 and 25 PSU, and the average within these treatments ranged from 332 and 352 mOsm kg^-1^. However at 30 PSU, the blood plasma osmolality had increased to 372 mOsm kg^-1^, significantly higher than in the 0–20 PSU treatments (Mann-Whitney U tests, p < 9.743 * 10^−4^) (Fig 3).

There was a significantly negative relationship between osmotic potential (blood plasma osmolality minus ambient osmolality) and AS (Spearman Rank Correlation, *r*_*s*_ = -1, *n* = 6, *p* < 0.01).

## Discussion

**Fig 4:**
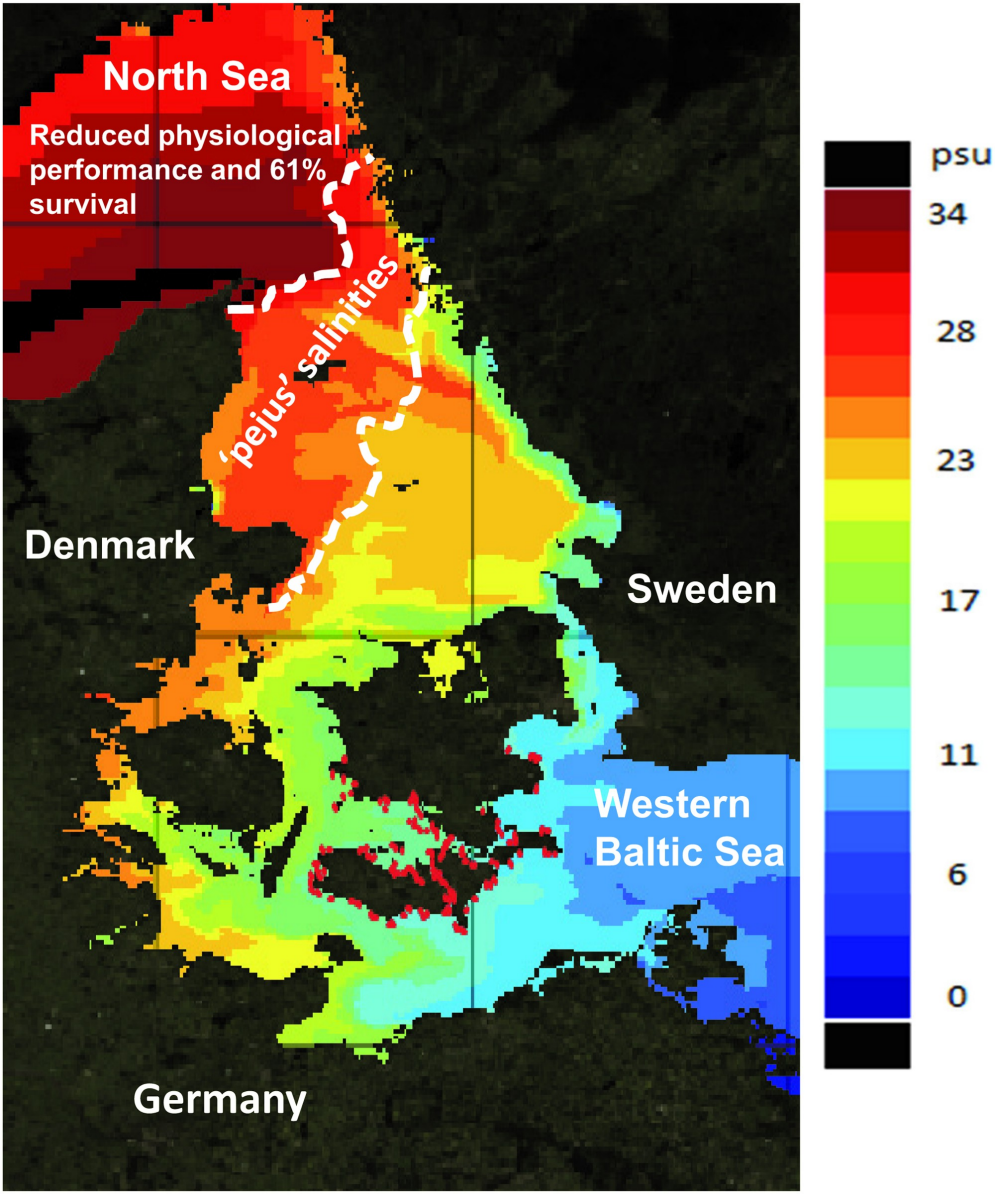
The salinity gradient between the brackish Western Baltic Sea and the full oceanic North Sea, with expected physiological performance and survival of round goby in relation to the ambient salinity. Salinities between approximately 20 and 25 PSU are considered ‘pejus’ salinities, i.e. where things are getting worse, whereas performance is significantly reduced at salinities of 25 PSU and above. Present occurrence (until 2013) of round goby is shown with red dots (modified from Azour et al 2015 [48]).

Maintaining physiological performance in novel environments is the most fundamental constraint potentially limiting dispersal of invasive species. The results support our prior expectation and show that the AS of round goby was significantly reduced at the highest salinities (25 and 30 PSU). The same pattern was seen for the osmoregulatory capacity, where round goby were able to maintain stable blood plasma osmolality up to 25 PSU, but had increased blood plasma osmolality at 30 PSU, indicating that 25 PSU is the species’ limit for unperturbed osmoregulation. Nevertheless, there was fairly high survival (i.e. 61%) of round goby at 30 PSU (Fig 4), and some individuals were able to maintain low blood plasma osmolality at the high salinities.

The present reduced AS is the outcome of the combined effects of increasing SMR at salinities departing from 10 PSU, and MMR following the inverse pattern, being reduced at 25 and 30 PSU. The augmented SMR likely reflects the additional costs of maintaining internal homeostasis, governing not only ion transport *per se*, but all factors related to this, including hormone level change and control and also potential underlying stress responses at the more extreme salinities [31, 49–51]. The gills are the principal organs of osmoregulation, but because of their involvement in respiration, they are at the same time the main site of water and ion leakage [30]. The reduced MMR observed at the two highest salinities is thus likely caused by reduced diffusion potential of the gill epithelium, initiated to reduce the osmotic water loss, which then simultaneously reduces the ability to take up oxygen (the osmo-respiratory compromise) [29, 30]. In combination, this agrees well with the present significant negative correlation between AS and osmotic potential, suggesting the latter to be one (of likely several) determining factor on AS.

Using AS to predict future species distribution during climate warming has gained much attention due to the oxygen and capacity limiting thermal tolerance (OCLTT) hypothesis [25, 52], which is an extension of the Fry Paradigm [20, 27]. The OCLTT hypothesis claims that AS is highest at an individual’s thermal optimum and drops as individuals approach their thermal limit, the latter caused by a failure of the fishes capacity to supply oxygen to tissues at temperature extremes [25, 52]. In spite of recent questioning of the general applicability of the OCLTT hypothesis [53–55], the results of the present study adhere to the Fry Paradigm and demonstrate impairment of oxygen transport systems when salinities exceed those which are optimal for AS. Thus, AS might be useful when evaluating distribution patterns of invasive fish species though salinity gradients. With the present substantial survival (61%—and the survivors obviously tolerating an increased plasma osmolality) and a mere 30% reduction of AS at the highest salinity (30 PSU), the question is whether the repressed physiological performance will hinder successful northward invasion into the full oceanic North Sea. Although growth potential and ability to compete with native species for resources could be affected in this high-saline environment [50, 56], it may be that 70% of the round goby’s current AS is sufficient to allow a population to establish there.

Previous studies on adult round goby with fresh water origin (Laurentian Great Lakes) have shown poor or no survival at salinities of 20 PSU or higher. In the study by Karsiotis et al. [57] all individuals died within 1 month at salinities of 25 and 30 PSU, and only 5% survived at 20 PSU, while Ellis and MacIsaac [58] showed a mortality of 100% after 48h at 30 PSU (only salinity tested). This is in stark contrast to the present study showing extensive survival of fish after three months at 30 PSU. The round goby in our study originated from Baltic brackish water, and thus the origin of the fish (fresh versus brackish waters) seemingly affects the species' ability to cope with salinity stress. Furthermore, Ellis and MacIsaac [58] and Karsiotis et al. [57] used acute exposure or very rapid salinity acclimation (up to 10 PSU h^-1^) in contrast to our study, where the salinity increase was 5 PSU week ^-1^. In Adriatic sturgeon acclimation to brackish water prior to an increasing salinity challenge test enabled the fish to maintain stable blood plasma osmolality [51], apparently by stimulating a beneficial increase in the activity of ion-translocating enzymes in the gills [59].

Gradual activation of various physiological functions (cardiac, metabolic and locomotor) in extreme environments has been shown in both aquatic and terrestrial animals [14, 60–65], and, together with the present results, point to the need to take acclimation into account when trying to understand and predict NIS performance in novel environments.

In addition to acclimation, several mechanisms exist by which physiological constraints may be overcome, hence enabling dispersal of individuals into and through unfavorable environments. It is now widely recognized that epigenetically induced changes in gene expression (i.e. changes that do not involve modification of DNA sequences) can evolve from a number of environmental stressors which then again determine how susceptible or resilient the individual is to specific stressors later in life ([66] and references herein). Likewise, maternal effects may act as adaptive mechanisms by buffering effects of a changing climate and thereby helping to alleviate some of the related fitness consequences [67, 68]. Furthermore, variations in the early rearing environment of genetically similar individuals can result in distinctively different physiological and behavioural responses when subject to stressors, thus enabling some individuals to cope better than others [69, 70]. Notably, in our study, some individuals were able to maintain unperturbed blood plasma osmolality levels at 25 and 30 PSU, resulting in high between-individual variation within these groups. Although our study was not designed to test for consistency in individual responses, these between-individuals differences may reflect variation in plasticity of the salinity tolerance, suggesting the existence of individuals with superior performance in high saline environments. Recent studies on fish have demonstrated how inter-individual differences in AS are associated with fitness-related components like dominance, position of individuals within schools, feeding capacity and growth [71–74]. Future studies targeting potential differences between individuals in salinity tolerance are needed to shed light on why some round goby appear to be more robust to environmental changes as these may be successful in relation to invasion of the North Sea.

### Mechanistic Species Distribution Models (SDMs)

The potential value of statistical species distribution models (SDMs) to describe species distributions has been demonstrated (e.g. [75]), but the predictive capability of these models has also be disputed, as they merely describe patterns in past time [76]. Mechanistic SDMs, parametised with physiological traits, are more robust when extrapolating distributions to future environmental conditions [76–79]. The critical issue is, however, which traits to incorporate into the SDM. Behavioral traits related to habitat preference [75], aggressiveness [80], or generalist vs. specialist strategies [81] may be relevant. However, the importance of behavioral traits may vary even on a short time scale (i.e. year-to-year), if the magnitude of competition or predation pressure fluctuates. Suboptimal habitats may not prevent colonization. For example, the round goby prefers relatively shallow protected areas of rocky substrate, but has been observed at water depth >20 m (own observations) and also appears to be thriving on mud bottom with only few rocks [82] and in tributaries [75]. The scope and applicability of the OCLTT hypothesis is under debate, yet many studies suggest that AS provides a more robust predictor of the ultimate geographical distribution [25, 83], as it links environmental properties directly to the physiological performance of the individual, including growth [73, 74, 84] and swimming performance [71, 85, 86] and indirectly via biotic interactions [87]. For example, Marras et al. [87] demonstrated that the relationship between temperature and AS provides a trait-based tool to evaluate the potential invasiveness of a NIS across temperature gradients.

Physiological constraints can only be overcome through evolutionary adaptation, epigenetics, maternal effects and/or effects of the early rearing environment (see above), and this could potentially be incorporated into mechanistic SDMs as researchers are beginning to develop an understanding of the processes underpinning local adaptations in non-static, non-equilibrium scenarios such as invasions across environmental gradients [79, 88, 89].

### Concluding remarks

In summary, our results suggest that physiological traits should be considered when evaluating the potential for dispersal of invasive species in novel environments. We found that salinity above 25 PSU causes a decrement in AS as well as a concomitant perturbation of plasma osmolality. This reduced physiological performance at the highest salinities may affect growth and competitive ability under oceanic conditions [50, 56], but to what extent reduced AS and osmoregulatory capacity will slow the current 30 km year^-1^ rate of advance of the species [48] through the steep salinity gradient from the brackish Baltic Sea and into the oceanic North Sea remains speculative. An unintended natural experiment is in progress to test whether the rate of advance slows down. At the current rate of advance the species will reach the oceanic North Sea by 2018/2019, therefore time for taking preventative action is short.

## Materials and methods

Ethical permit 2015-15-0201-00546 from the Danish Animal Ethics Committee covered all experiments reported here. Fish were observed twice daily by animal caretakers, the authors or the veterinarian at the aquarium. Humane endpoints were used, i.e. fish that showed signs of suffering or distress (ceasing of feeding, loss of equilibrium, increased ventilation frequency, and lack of normal movement and social interactions) were euthanized by a sharp blow to the head where after the head was cut off. Three weeks into the acclimatization protocol (i.e. when one group of fish was at 0 PSU, one group at 10 PSU and the remaining four groups at 15 PSU) necropsy of two euthanized fish revealed heavy infection (skin and gills) with Trichodina sp. Individual fish in all tanks were subsequently given a 20min freshwater bath [90, 91] after which they were returned to their acclimation tank. Regular samplings of skin scrapes throughout the remaining experimental period did not reveal any further Trichodina sp. infections, and no signs of gill lesions/gill abnormalities were seen. From this, and also considering the short period of infection, it was assumed that Trichodina sp. had not affected the capacity for either oxygen uptake or ion exchange at the time when data collection commenced. For blood sampling for measurements of blood plasma osmolality, the fish were anaesthetized (see below for details).

### Fish and experimental setup

Round gobies (approximately 200 fish) were caught with fyke nets in the brackish water estuary Guldborgsund (salinity 10 PSU [92]) in the western Baltic Sea (54°42’N, 11°51’E). They were transported to Den Blå Planet National Aquarium Denmark, Kastrup, Denmark, and held in freshwater (0 PSU) for 3 months until the experimental acclimation began. Fish were fed to satiation three times a week with 1.5–4 mm commercial high-nutrition fish feed pellets on which they fed well after 1–2 weeks of customization. Fresh- instead of brackish water was chosen due to practical reasons, as the species thrives well in fresh water (own previous experience; [93]). Altogether 100 adult fish of comparable size were allocated into five separate acclimation tanks containing 450 L 10 PSU water, with 20 fish in each tank. After one week at 10 PSU, the salinity was increased gradually by 5 PSU per week to obtain treatment salinities of 10, 15, 20, 25 and 30. Thus, the 10 PSU group were already at their final treatment salinity, the 15 PSU had reached their final treatment salinity after one week, the 20 PSU group after two weeks, the 25 PSU group after three weeks, while fish in the 30 PSU group had reached their final salinity after four weeks. A remaining batch of 20 fish, of comparable size to the above-mentioned 100 fish, was kept in the fresh water, i.e. constituting the 0 PSU group. Once each group of fish reached their experimental salinity, they were kept at this salinity for 8 days and then blood was sampled for determination of blood plasma osmolality. Subsequently, the fish were left for a between 12 and 20 days before respirometry (for further details of experiments see below). Survival was determined by summing the number of dead fish in each treatment group over the entire period, i.e. from the start, when fish were separated into the tanks and acclimated to the different salinities until the end of all experiments. Plastic pipes and artificial seaweed were used as shelter structures for the fish in all tanks. The feeding regime continued as described above, and there were no signs of feeding ceasing at any point of time in any group of fish. Brackish water was obtained by mixing filtered oceanic sea water (30 PSU) and non-chlorinated tap water (0 PSU) and salinity measured with a digital refractometer (Pocket refractometer PAL-06S from Atago). Water temperature was 15–17°C, and the daylight period held at 9 h. To maintain adequate water quality, the water in each tank was recirculated through 20 L canister filters with a capacity of 3500 L h^-1^ and 50% exchanged twice a week. Water quality was measured once a week, ammonium (NH_4_^+^) and nitrite (NO_2_^-^) levels with a spectrophotometer (7500 Photometer, Palintest instruments Ltd, Gateshead, UK) and pH with a hand held pH meter (HQ30d Portable pH Meter, Hach, Loveland, US).

### Respirometry

Forty-eight fish (n = 8 in each group) were used for respirometry (Table 1).

**Table 1:** Size (mean ± SD) of round goby used in respirometry and for blood plasma osmolality experiments. Length is total length, and Fulton’s condition factor (K) was calculated as K = 100×W×L^-3^ (where W is in g and L is total length in cm).

	Treatment salinity (PSU)					
	0	10	15	20	25	30
	Fish for respirometry					
N	8	8	8	8	8	8
Length (mm)	152 ± 11	154 ± 11	154 ± 16	157 ± 11	149 ± 9	148 ± 11
Weight (g)	53 ± 12	53 ± 13	56 ± 17	54 ± 11	45 ± 8	45 ± 12
Fultons K	1.48 ± 0.1	1.42 ± 0.17	1.50 ± 0.11	1.38 ± 0.07	1.37 ± 0.15	1.37 ± 0.15
	Fish for blood plasma osmolality					
N	10	9	10	10	10	9
Length (mm)	160 ± 22	161 ± 11	163 ± 20	166 ± 16	162 ± 15	154 ± 12
Weight (g)	66 ± 26	60 ± 14	65 ± 20	60 ± 18	57 ± 17	53 ± 15
Fultons K	1.56 ± 0.11	1.41 ± 11	1.46 ± 0.10	1.29 ± 0.10	1.34 ± 0.12	1.42 ± 0.08

They were kept for a minimum of 20 days at their final treatment salinity before respirometry experiments were initiated, a time period considered sufficient for their blood plasma osmolality level to be restored after the salinity change [49, 94, 95]. Individuals were fasted for two days before being placed in the respirometer. Four respirometers with volumes between 1126 and 1157 mL were held in an 80 L aerated, temperature-regulated water bath (18°C, the range throughout the study was± 0.5°C, and within one trial ± 0.25°C), placed at an undisturbed site. The water was exchanged once a week. To minimize visual disturbance, the respirometers were shielded off from each other and from above with non-transparent polyethylene plates. Oxygen consumption rate (ṀO_2_) was measured with an intermittent-flow respirometry set-up [96, 97]. The partial pressure of oxygen (pO_2_) was measured every second with fiber-optic sensors, connected to a Witrox 4 oxymeter (Loligo Systems, Tjele, Denmark). Using the software AutoResp^™^ (Loligo Systems, Tjele, Denmark) $\frac{dpO2}{dt}$ (kPaO_2_ h^-1^) was determined by linear regression and ṀO_2_ (mg O_2_ kg^-1^ h^-1^) calculated according to the equation:

$$\overset{´}{M}O_{2}=\frac{\alpha \cdot V_{resp}\cdot \beta }{BM}$$ (4)

where α is the $\frac{dpO2}{dt}$, V_resp_ is the total volume of the respirometer minus the volume of the fish (L), *β* is oxygen solubility at the given temperature and salinity and BM is the body mass of the fish (kg). The animal volume was assumed equal to its mass.

Whole animal oxygen consumption rate (ṀO_2,whole animal_; mg O_2_ h^-1^) scales allometrically with BM over very large BM ranges [98], yet with narrow BM ranges one can assume isometric scaling. To investigate whether BM in the present study was taken appropriately into account in the calculation of ṀO_2_ (Eq 1), simple linear regressions analyses (ṀO_2,whole animal_ = a*BM + b) were conducted, one for MMR yielding an R^2^ of 0.567 (F(1,46) = 62.554, p = 4.028*10^−10^) and one for SMR yielding and R^2^ of 0.698 (F(1,46) = 106.212, p = 1.547*10^−13^). This supported that ṀO_2,whole animal_ here could be assumed to scale isometrically with BM, justifying using Eq 4.

Before and after each trial, background ṀO_2_ was measured in the empty respirometers using a wait period of 400 s and a measurement period of 3600 s. To yield maximum metabolic rate (MMR), individual fish were manually exercised to exhaustion (between 3–5 min) in a circular tank (40 cm diameter, water depth 10 cm) [99], after which the fish was placed in the respirometer. The time until the experiment began was approximately 60 s, and the first ṀO_2_ measurement taken with wait period of 60 s and a measuring period of 150 s. The background ṀO_2_ from before the trial was subtracted from this initial ṀO_2_ value, which was then considered MMR [99, 100].

**Fig 5:**
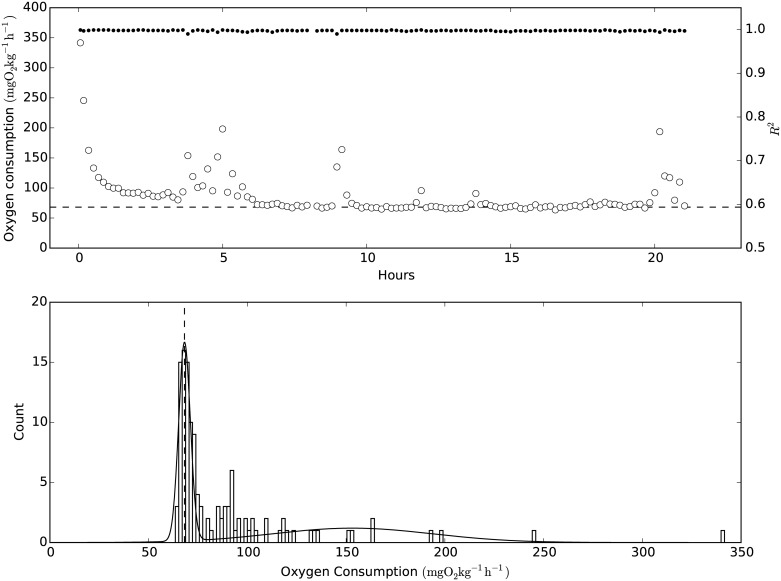
An example of the oxygen consumption rate during an experiment and the determination of standard metabolic rate. Upper panel: Open circles represent oxygen consumption rate (ṀO_2_) and solid circles represent the corresponding R^2^. The values were recorded at 25 PSU and 18°C. Lower panel: a histogram of all ṀO_2_ values with an R^2^ above 0.95, fitted to a double Gaussian distribution (solid line). The standard metabolic rate is shown in both panels by punctuated lines, and was determined as the lowest mean of the two Gaussian distributions.

After measurement of MMR, fish remained in the respirometers overnight to allow for ṀO_2_ measurement of the individual at a resting, non-digesting state (standard metabolic rate; SMR [81]) (flush period of 240 s, wait period of 60 s, measuring period of 300 s). A single experimental trial lasted approximately 22 h. The applied measurement periods yielded almost exclusively regression with R^2^ > 0.99, and the oxygen content never fell below 70%. ṀO_2_ values with R^2^ < 0.95 were removed before data processing, and a double Gaussian distribution was fitted to the frequency distribution of the ṀO_2_ values from the individual fish (Fig 5).

This algorithm separates the ṀO_2_ associated with spontaneous activity and handling stress from those of the resting animal, and SMR was determined as the lowest mean value of the two Gaussian distributions [101].

Background ṀO_2_ from before the trial was subtracted from MMR measurements, whereas the value after the trial was subtracted from the SMR measurements. Aerobic scope (AS) was expressed as MMR minus SMR. The background ṀO_2_ was 1% of MMR measurements and 6% of SMR measurements (calculated on global averages).

### Blood plasma osmolality

The fish (n = 9–10 in each group, Table 1) were kept for eight days at their final treatment salinity before blood sampling, a time period considered sufficient for their blood plasma osmolality level to be restored after the salinity change [49, 94, 95]. Following blood sampling fish were returned to their tank, and a randomised sample of these fish was used a minimum of 12 days later for respirometry. The 12 day period was deemed long enough to restore blood hematocrit, hemoglobin and osmolality [102, 103]. The fish were anaesthetized in a 0.133 g L^-1^ 3-aminobenzoicacid ethyl ester (MS-222) solution buffered with sodium bicarbonate (0.30 g l^-1^) [104] and blood subsequently sampled by caudal puncture with lithium-heparinized 27 gauge hypodermic needle [105]. The samples (approximately 200 μL) were immediately spun at 6000 rpm for 5 min, and plasma isolated and stored in a -18°C fridge. Osmolality was subsequently measured in a vapour pressure point osmometer (Vapor Pressure Osmometer 5600, Wescor, South Logan, USA).

### Statistical analyses

Statistics were performed in the software SPSS statistics 23 (IBM, Armonk, USA) and R [106] using a significance level (α) of 0.05. There was variance homogeneity for MMR, SMR, and AS (Levene’s test, all p > 0.147), and normality for the data in all treatments (Shapiro-Wilk’s tests, p > 0.102) except for SMR at a salinity of 25 (Shapiro-Wilk’s test, p = 0.048). This single occurrence of non-normality was ignored since ANOVAs are relatively robust towards non-normality [107].

A general linear model was used to conduct one-way ANOVAs to test the effect of salinity on SMR, MMR and AS. The models also tested if the 0, 15, 20, 25, and 30 PSU treatments produced SMR, MMR, and AS significantly different from 10 PSU (the reference level), which is the condition nearest isoosmotic levels, as well as the environmental salinity in Guldborgsund where the round goby were caught. It is well known that some individuals are extremely difficult to exercise to exhaustion (i.e. to achieve true measurements of MMR) using standard tests [21]. Hence, to avoid confounding of the analysis by abnormal behaviour, we excluded data if the MMR value was found to be outside 2 standard deviations of the mean calculated for the remaining 7 data points in the treatment group (i.e. outside the 95% confidence interval). This resulted in the removal of two outliers (one from 10 PSU and one from 15 PSU). Since MMR constitutes a part of AS, the two individuals identified as outliers on MMR, were also disqualified for AS, but their SMR were still included in the analysis. The model also parameterized the response in relation to a predefined set of parameters, given as:

$$MO_{2}=a*SAL+b*SAL^{2}+c$$ (5)

where a,b and c are constants to be estimated and SAL is salinity. The second order term was included, as local maxima and minima were expected.

The osmolality data was log transformed to obtain variance homogeneity (Levene’s test, p = 0.114), but the transformation did not yield normality at a salinity of 25 (Shapiro-Wilk’s tests, p = 0.025) and 30 (Shapiro-Wilk’s tests, p = 0.002). Therefore, a Kruskal-Wallis’ test was applied, followed by pairwise Mann-Whitney U tests with a Bonferroni correction for multiple comparisons (Bonferroni’s α = 0.003).

A Spearman Rank Correlation was carried out between the average osmotic potential for each treatment ([blood plasma osmolality minus ambient osmolality]) and average for each treatment AS.

## Supporting information

S1 Table Data on oxygen uptake and blood plasma osmolality. All individual values of standard metabolic rate (SMR), maximum metabolic rate (MMR) and aerobic scope (AS), in addition to blood plasma osmolality and osmotic gradient, are shown in relation to ambient (treatment) salinity.
(PDF)

## References

[1] Bax N, Williamson A, Aguero M, Gonzalez E, Geeves W. Marine invasive alien species: a threat to global biodiversity. Mar Pol. 2003;27: 313–323.

[2] Simberloff D. How common are invasion-induced ecosystem impacts? Biol Invasions. 2011;13: 1255–1268.

[3] Katsanevakis S, Wallentinus I, Zenetos A, Leppäkoski E, Çinar ME, Oztürk B, et al Impacts of invasive alien marine species on ecosystem services and biodiversity: a pan-European review. Aquat Inv.2014; 9: 391–423.

[4] Galil BS, Marchini A, Occhipinti-Ambrogi A, Minchin D, Narščius A, Ojaveer H, Olenin S. International arrivals: widespread bioinvasions in European Seas. Ethol Ecol & Evol. 2014;26: 152–171. 10.1080/03949370.2014.897651 PMC4034525 24899770

[5] Ojaveer H, Galil BS, Minchin D, Olenin S, Amorim A, Canning-Clode J, et al Ten recommendations for advancing the assessment and management of nonindigenous species in marine ecosystems. Mar Policy 2014;44: 160–165.

[6] Olenin S, Narščius A, Minchin A, David M, Galil B, Gollasch S, et al Making non-indigenous species information systems practical for management and useful for research: an aquatic perspective. Biol Cons. 2014;173: 98–107.

[7] Lehtiniemi M, Ojaveer H, David M, Galil B, Gollasch S, McKenzie C, et al Dose of truth—monitoring marine nonindigenous species to serve legislative requirements. Mar Policy 2015;54: 26–35.

[8] Ojaveer H, Kotta J. Ecosystem impacts of the widespread non-indigenous species in the Baltic Sea: literature survey evidences major limitations in knowledge. Hydrobiologia 2015;750: 171–185.

[9] Ojaveer H, Galil BS, Lehtiniemi M, Christoffersen M, Clink S, Florin A-B, et al Twenty five years of invasion: management of the round goby *Neogobius melanostomus* in the Baltic Sea. Man Biol Inv. 2015; 6: 329–339.

[10] Ojaveer H, Galil BS, Campbell ML, Carlton JT, Canning-Clode J, Cook EJ, et al Classification of Non-Indigenous Species Based on Their Impacts: Considerations for Application in Marine Management. PLoS Biol 2015;13(4): e1002130 10.1371/journal.pbio.1002130 25875845 PMC4398364

[11] Child T, Benjamin BI, Shine R. Abiotic and Biotic influences on the dispersal behavior of metamorph cane toads (*Bufo marinus*) in tropical Australia. J Exp Zool. 2008;309A: 215–224. 10.1002/jez.450 18288694

[12] Beauchamp DA. Bioenergetic ontogeny: linking climate and mass specific feeding to life-cycle growth and survival of salmon. Am Fish Soc Symp. 2009;70: 1–19.

[13] Pörtner HO, Peck MA. Climate change effects on fishes and fisheries: towards a cause-and-effect understanding. J Fish Biol. 2010;77: 1745–1779. 10.1111/j.1095-8649.2010.02783.x 21078088

[14] Seebacher F, Franklin CE. Physiology of invasion: cane toads are constrained by thermal effects on physiological mechanisms that support locomotor performance. J Exp Biol. 2011;214: 1437–1444. 10.1242/jeb.053124 21490252

[15] Gifford ME, Kozak KH. Islands in the sky or squeezed at the top? Ecological causes of elevational range limits in montane salamanders. Ecography 2012;35: 193–203.

[16] Williamson M. Biological invasions. New York, Chapman & Hall; 1997

[17] Mack RN, Simberloff D, Lonsdale WM, Evans H, Clout M, Bazzaz FA. Biotic invasions: causes, epidemiology, global consequences, and control. Ecol Appli. 2000;10: 689–710.

[18] Crooks J, Rilov G. The establishment of invasive species In: Crooks J, Rilov G, editors. Biological invasions in marine ecosystems: ecological, management and geographic perspectives. Springer-Verlag: Berlin; 2009 pp. 173–175.

[19] Lennox R, Choi K, Harrison PM, Paterson JE, Peat TB, Ward TD, Cooke SJ. Improving science-based invasive species management with physiological knowledge, concepts, and tools. Biol Invasions 2015;17: 2213–2227.

[20] Fry FEJ. Effect of the environment on animal activity. Univ. Toronto Stud. Biol. Ser. 1947;55: 1–62.

[21] Norin T, Clark TD. Measurement and relevance of maximum metabolic rate in fishes. J Fish Biol. 2016;88: 122–151. 10.1111/jfb.12796 26586591

[22] Chabot D, Steffensen JF, Farrell AP. The determination of standard metabolic rate in fishes. J Fish Biol. 2016;88: 81–121. 10.1111/jfb.12845 26768973

[23] Waversveld Van J, Addink ADF, Van Den Thillart G, Smit H. Heat production of fish: A literature review. Comp Biochem Physiol. A 1989;92: 159–162.,

[24] Ej Eliason, Farrell AP. Oxygen uptake in Pacific salmon *Oncorhynchus* spp.: when ecology and physiology meet. J Fish Biol. 2015; 10.1111/jfb.12790 26577675

[25] Pörtner HO, Farrell AP. Physiology and Climate Change. Science 2008;322: 690–692. 10.1126/science.1163156 18974339

[26] Tirsgaard B, Berens J, Steffensen JF. The effect of temperature and body size on metabolic scope of activity in juvenile Atlantic cod *Gadus morhua* L. Comp Biochem Physiol. A 2015;179: 89–94. 10.1016/j.cbpa.2014.09.033 25281351

[27] Fry FEJ. The effects of environmental factors on the physiology of fish In: Hoar WS, Randall DJ, editors. Fish Physiology. New York, NY: Academic Press; 1971 pp. 1–98.

[28] Hettler WF. Influence of temperature and salinity on routine metabolic rate and growth of young Atlantic menhaden. J Fish Biol. 1976;8: 55–65.

[29] Randall DJ, Baumgarten D, Malyusz M. The relationship between gas and ion transfer across the gills of fishes. Comp BiochemPhysiol. A;41: 629–637. 10.1016/0300-9629(72)90017-5 4401733

[30] Nilsson S. Control of gill blood flow In: Nilsson S, Holmgren S, editors. Fish physiology. Croom Helm: London; 1986 pp 87–101.

[31] Kültz D. Physiological mechanisms used by fish to cope with salinity stress. J Exp Biol. 2015;218: 1907–1914. 10.1242/jeb.118695 26085667

[32] Lutz PL. Ionic and body compartment responses to increasing salinity in the perch *Perca fluviatilis*. Comp Biocehm Physiol. A 1972;42: 711–717. 10.1016/0300-9629(72)90449-5 4404267

[33] Jude DJ, Reider RH, Smith GR. Establishment of Gobiidae in the Great Lakes basin. Can J Fish Aquat Sci. 1992;49: 416–421.

[34] Sapota MR, Skora KE. Spread of alien (non-indigenous) fish species *Neogobius melanostomus* in the Gulf of Gdánsk (south Baltic). Biol Invasions 2005;7: 157–164.

[35] Borcherding J, Staas S, Krüuger S, Ondraĉková M, Šlapanský L, Jurajda P. Non-native Gobiid species in the lower River Rhine (Germany): recent range extensions and densities. J Appl Ichth. 2011;27: 153–155.

[36] Kotta J, Nurkse K, Puntila R, Ojaveer H. Shipping and natural environmental conditions define the distribution of the invasive non-indigenous round goby *Neogobius melanostomus* in a regional sea. Estuar Coast Shelf Sci. 2015;169: 15–24.

[37] Bronnenhuber JE, Dufour BA, Higgs DM, Heath DD. Dispersal strategies, secondary range expansion and invasion genetics of the nonindigenous round goby, *Neogobius melanostomus*, in Great Lakes tributaries. Mol Ecol. 2011;20: 1845–1859. 10.1111/j.1365-294X.2011.05030.x 21492265

[38] Gutowskya LFG, Brownscombea JW, Fox MG. Angling to estimate the density of large round goby (*Neogobius melanostomus*). Fish Res. 2011;108: 228–231.

[39] LaRue EA, Ruetz CR, Stacey MB, Thum RA. Population genetic structure of the round goby in Lake Michigan: implications for dispersal of invasive species. Hydrobiologia 2011;663: 71–73.

[40] Lauer TE, Allen PJ, McComish TS. Changes in mottled sculpin and johnny darter trawl catches after the appearance of round gobies in the Indiana waters of Lake Michigan. Trans Am Fish Soc. 2004;133: 185–189.

[41] Sapota MR. The round goby (*Neogobius melanostomus*) in the Gulf of Gdánsk—a species introduction into the Baltic Sea. Hydrobiologia 2004;514: 219–224.

[42] Balshine S, Verma A, Chant V, Theysmeyer T. Competitive interactions between round gobies and logperch. J Great Lakes Res. 2005;31: 68–77.

[43] Fitzsimons JD, Williston B, Fodor G, Bravener G, Jonas JL, Claramunt RM, et al Laboratory estimates of salmonine egg predation by round gobies (*Neogobius melanostomus*), sculpins (*Cottus cognatus* and *C*. *bairdi*) and crayfish (*Orconectes propinquus*). J Great Lakes Res. 2006;32: 227–241.

[44] Karlson AML, Almqvist G, Skora KE, Appelberg M. Indications of competition between non indigenous round goby and native flounder in the Baltic Sea. ICES J Mar Sci. 2007;64: 479–486.

[45] Lederer AM, Janssen J, Reed T, Wolf A. Impacts of the introduced round goby (*Apollonia melanostoma*) on Dreissenids (*Dreissena polymorpha* and *bugensis*) and on macroinvertebrate community between 2003 and 2006 in the littoral zone of Green Bay, Lake Michigan. J Great Lakes Res. 2008:34; 690–697.

[46] Flink H. Is the boldness of invasive gobies a predictor of individual ability to outcompete native species? M.Sc. Thesis, Linnæus University. 2015. http://lnu.diva-portal.org/smash/record.jsf?pid=diva2%3A755157&dswid=1000

[47] Millero FJ, Chetirkin PV. The density of Caspian Sea waters. Deep-Sea Res. 1980;27A: 265–271.

[48] Azour F, van Deurs M, Behrens J, Carl H, Hüssy K, Greisen K, et al Invasion rate and population characteristics of the invasive round goby *Neogobius melanostomus*: effects of density and invasion history. Aquat Biol 2015;24: 41–52.

[49] Morgan JD, Iwama GK. Salinity effects on oxygen consumption, gill Na+,K+-ATPase activity and ion regulation in juvenile coho salmon. J Fish Biol. 1998;53: 1110–1119.

[50] Boeuf G, Payan P. How should salinity influence fish growth? Comp Biochem Physiol. C *See comment in PubMed Commons below* 2001;130: 411–23. 10.1016/s1532-0456(01)00268-x 11738629

[51] McKenzie DJ, Cataldi E, Romano P, Owen SF, Taylor EW, Bronzi P. Effects of acclimation to brackish water on the growth, respiratory metabolism, and swimming performance of young-of-the-year Adriatic sturgeon (*Acipenser naccarii*). Can J Fish Aquat. Sci. 2001;58: 1104–1112.

[52] Pörtner HO. Climate change and temperature-dependent biogeography: oxygen limitation of thermal tolerance in animals. Naturwissenschaften 2001;88: 137–146. 11480701 10.1007/s001140100216

[53] Clark TD, Sandblom E, Jutfelt F. Aerobic scope measurements of fishes in an era of climate change: respirometry, relevance and recommendations. J Exp Biol. 2013;216: 2771–82. 10.1242/jeb.084251 23842625

[54] Gräns A, Jutfelt F, Sandblom E, Jönsson E, Wiklander K, Seth H, Olsson C, Dupont S, Ortega-Martinez O, Einarsdottir I, Björnsson BT, Sundell K, Axelsson M. Aerobic scope fails to explain the detrimental effects on growth resulting from warming and elevated CO_2_ in Atlantic halibut. J Exp Biol. 2014;217: 711–717. 10.1242/jeb.096743 24574386

[55] Norin T, Malte H, Clark TD. Aerobic scope does not predict the performance of a tropical eurythermal fish at elevated temperatures. J Exp Biol. 2014;217: 244–251. 10.1242/jeb.089755 24115064

[56] Alcaraz C, Bisazza A, Garcıa-Berthou E. Salinity mediate the competitive interactions between invasive mosquitofish and an endangered fish. Oecologia 2008;155: 205–213. 10.1007/s00442-007-0899-4 17999091

[57] Karsiotis SI, Pierce LR, Brown JE, Stepien CA. Salinity tolerance of the invasive round goby: experimental implications for seawater ballast exchange and spread to North American estuaries. J Great Lakes Res. 2012;38: 121–128.

[58] Ellis S, MacIsaac HJ. Salinity tolerance of Great Lakes invaders. Freshw. Biol. 2009;54: 77–89.

[59] McKenzie DJ, Cataldi E, Di Marco P, Mandich A, Romano P, Ansferri S, Bronzi P, Cataudella S. Some aspects of osmotic and ionic regulation in Adriatic sturgeon (*Acipenser naccarii*). II. Morpho-physiological adjustments to hyperosmotic environments. J Appl Ichthyol. 1999;15: 61–66.

[60] St Pierre J, Charest P-M, Guderley H. Relative contribution of quantitative and qualitative changes in mitochondria to metabolic compensation during seasonal acclimatisation of rainbow trout *Oncorhynchus mykiss*. J Exp Biol 1998;201: 2961–2970.

[61] Hammill E, Wilson RS, Johnston IA. Sustained swimming performance and muscle structure are altered by thermal acclimation in male mosquitofish. J Therm Biol. 2004;29: 251–257.

[62] Franklin CE, Davidson W, Seebacher F. Antarctic fish compensate for rising temperatures: thermal acclimation of cardiac performance in *Pagothenia borchgrevinki*. J Exp Biol. 2007;210: 3068–3074. 10.1242/jeb.003137 17704081

[63] Kassahn KS, Crozier RH, Pörtner HO, Caley MJ. Animal performance and stress: responses and tolerance limits at different levels of biological organisation. Biol. Rev. Camb Philos Soc. 2009;84: 277–92. 10.1111/j.1469-185X.2008.00073.x 19344429

[64] Winwood-Smith HS, Alton LA, Franklin CE, White CR. Does greater thermal plasticity facilitate range expansion of an invasive terrestrial anuran into higher latitudes? Cons. Physiol. 2015;3:10.1093/conphys/cov010 PMC4778455 27293695

[65] Farrell AP, Franklin CE. Recognizing thermal plasticity in fish. Science 2016;351: 132–133. 10.1126/science.351.6269.132-b 26744399

[66] Nestler EJ. Transgenerational Epigenetic Contributions to Stress Responses: Fact or Fiction? PLOS Biology 2016; 10.1371/journal.pbio.1002426 PMC4807775 27015088

[67] Vehmaa A, Brutemark A, Engström-Öst J. Maternal Effects May Act as an Adaptation Mechanism for Copepods Facing pH and Temperature Changes. PLoS ONE 2012;7(10): e48538 10.1371/journal.pone.0048538 23119052 PMC3485336

[68] Shama LNS, Strobel A, Mark FC, Wegner KM. Transgenerational plasticity in marine sticklebacks: maternal effects mediate impacts of a warming ocean. Func Ecol. 2014;28: 1482–1493

[69] Salonius K, Iwama GK. Effects of Early Rearing Environment on Stress Response, Immune Function, and Disease Resistance in Juvenile Coho (*Oncorhynchus kisutch*) and Chinook Salmon (*O*. *tshawytscha*). Can J Fish Aquat Sci. 1993;50: 759–766.

[70] DePasquale C, Neuberger T, Hirrlinger AM, Braithwaite VA. The influence of complex and threatening environments in early life on brain size and behavior. Proc Biol Sci. 2016;27: 283(1823): 20152564 10.1098/rspb.2015.2564 PMC4795028 26817780

[71] Killen SS, Marras S, Steffensen JF, McKenzie DJ. Aerobic capacity influences the spatial position of individuals within fish schools. Proc Royal Soc B 2012;279: 357–64. 10.1098/rspb.2011.1006 PMC3223687 21653593

[72] Killen SS, Mitchell MD, Rummer JL, Chivers DP, Ferrari MOC, Meekan MG. et al Aerobic scope predicts dominance during early life in a tropical damselfish. Func Ecol. 2014;28: 1367–1376.

[73] Auer SK, Salin K, Rudolf AM, Anderson GJ, Metcalfe NB. Aerobic cope explains individual variation in feeding capacity. Biol Let. 2015;11: 20150793. 26556902 10.1098/rsbl.2015.0793 PMC4685545

[74] Auer S. K., Salin K., Rudolf A. M., Anderson G. J. and Metcalfe N. B. (2015) The optimal combination of standard metabolic rate and aerobic scope for somatic growth depends on food availability. Func. Ecol. 29, 479–486.

[75] Kornis MS, Vander Zanden MJ. Forecasting the distribution of the invasive round goby (*Neogobius melanostomus*) in Wisconsin tributaries to Lake Michigan. Can J Fish Aquat Sci. 2010;67; 553–562.

[76] Evans T. G., Diamond S. E. and Kelly M. W. (2015). Mechanistic species distributions modeling as a link between physiology and conservation. Cons. Physiol. 3, 10.1093/conphys/cov056 PMC4778482 27293739

[77] Peterson AT. Predicting the geography of species’ invasions via ecological niche modeling. Quart Rev Biol. 2003;78: 419–433. 14737826 10.1086/378926

[78] Buckley LB, Urban MC, Angilletta MJ, Crozier LG, Rissler LJ, Sears MW. Can mechanisms inform species’ distribution models? Ecol Letters 2010;13: 1041–1054. 10.1111/j.1461-0248.2010.01479.x 20482574

[79] Kearney M, Porter W. Mechanistic niche modelling: combining physiological and spatial data to predict species’ ranges. Ecol letters 2009;12: 334–350. 10.1111/j.1461-0248.2008.01277.x 19292794

[80] Duckworth RA, Badyaev AV. Coupling of dispersal and aggression facilitates the rapid range expansion of a passerine bird. Proceedings of the Natural Academy of Science 2007;104: 15017–15022. 10.1073/pnas.0706174104 PMC1986605 17827278

[81] Gilchrist GW. Specialists and generalists in changing environments. I. Fitness landscapes of thermal sensitivity. Am Nat. 1995;146: 252–270.

[82] Young JA, Marentette JR, Gross C, McDonald JI, Verma A, Marsh-Rollo E, et al Demography and substrate affinity of the round goby (*Neogobius melanostomus*) in Hamilton Harbour. J Great Lakes Res. 2010;36: 115–122.

[83] Pörtner HO, Knust R. Climate change affects marine fishes through the oxygen limitation of thermal tolerance. Science 2007;315: 95–97. 10.1126/science.1135471 17204649

[84] Claireaux G, Lefrançois C. Linking environmental variability and fish performance: integration through the concept of scope for activity. Phil Trans Royal Soc London B 2007;362: 2031–2041. 10.1098/rstb.2007.2099 PMC2442852 17472923

[85] Reidy SP, Kerr SR, Nelson JA. Aerobic and anaerobic swimming performance of individual Atlantic cod. J Exp Biol. 2000;203: 347–357. 10607544 10.1242/jeb.203.2.347

[86] Killen SS, Nati JJH, Suski CD. Vulnerability of individual fish to capture by trawling is influenced by capacity for anaerobic metabolism. Proc. Royal Soc. B 2015;282: 20150603. 10.1098/rspb.2015.0603 PMC4632608 26246542

[87] Marras S, Cucco A, Antognarelli F, Azzurro E, Milazzo M, Bariche M, et al Predicting future thermal habitat suitability of competing native and invasive fish species: from metabolic scope to oceanographic modelling. Cons Physiol. 2015; 3 10.1093/conphys/cou059 PMC4778460 27293680

[88] Phillips BL, Brown GP, Shine R. Life-history evolution in range-shifting populations. Ecology 2010;91: 1617–1627. 20583704 10.1890/09-0910.1

[89] Munday PL. Evolutionary ecology: Survival of the fittest. Nat. Climate Change 2015;5: 102–103.

[90] Lom J. Protozoan and Metazoan Infections In: Woo PTK, editor. Fish Diseases and Disorders. CABI Publishing: New York; 1995 Volume 1.

[91] Smith SA, Noga EJ. General Parasitology In: Stoskopf MK, editor. Fish Medicine. W.B. Saunders Co.: Philadelphia; 1992.

[92] Olsen JS. Vækst, migration og reproduktion hos en dansk population af brakvandsaborre (Perca fluviatilis L.). M. Sc. Thesis, The University of Copenhagen. 2002. http://site.vordingborg.dk/Everest/Publications/Afdelinger/Fagsekretariat%20Natur/20080310142639/CurrentVersion/Brakvandsaborre%20speciale.pdf

[93] Kornis MS, Mercado-Silva N, Vander Zanden MJ. Twenty years of invasion: a review of round goby (*Neogobius melanostomus*) biology, spread and ecological implications. J Fish Biol. 2012;80: 235–285. 10.1111/j.1095-8649.2011.03157.x 22268429

[94] Hwang PP, Sun CM, Wu SM. Changes of plasma osmolality, chloride concentration and gill Na-K-ATPase activity in tilapia *Oreochromis mossambicus* during seawater acclimation. Mar Biol. 1989;100: 295–299.

[95] Van der Linden A, Vanaudenhove M, Verhoye M, De Boeck G, Blust R. Osmoregulation of the common carp (*Cyprinus carpio*) when exposed to an osmotic challenge assessed in-vivo and non-invasively by diffusion- and T2-weighted magnetic resonance imaging. Comp. Biochem. Physiol. A 1999;124: 343–352.

[96] Steffensen JF, Johansen K, Bushnell PG. An automated swimming respirometer. Comp Biochem Physiol A 1984;79: 437–440.

[97] Svendsen MBS, Bushnell PG, Steffensen JF. Design and setup of intermittent-flow respirometry system for aquatic organisms. J Fish Biol. 2016;88: 26–50. 10.1111/jfb.12797 26603018

[98] Clarke A, Johnston NM. Scaling of metabolic rate with body mass and temperature in teleost fish. J Anim Ecol. 1999;68: 893–905

[99] Behrens JW, Steffensen JF. The effect of hypoxia on behavioural and physiological aspects of lesser sandeel, *Ammodytes tobianus* (Linnaeus, 1785). Mar Biol. 2007;150: 1365–1377

[100] Reidy SP, Nelson JA, Tang Y, Kerr SR. Post-exercise metabolic rate in Atlantic cod and its dependence upon the method of exhaustion. J Fish Biol. 1995;47: 377–386.

[101] Steffensen JF, Bushnell PG, Schurmann H. Oxygen consumption in four species of teleosts from Greenland: no evidence of metabolic cold adaptation. Polar Biol. 1994;14: 49–54.

[102] Soivio A, Nyholm K. A technique for repeated sampling of the blood of individual resting fish. J Exp Biol. 1975;62:, 207–2017 10.1242/jeb.63.1.207 1159362

[103] Soivio A, Nyholm K, Huhti M. Effects of anaesthesia with MS222, neutralized MS 222 and benzocaine on the blood constituents of rainbow trout, *Salmo gairdneri*. J Fish Biol. 1977;10: 91–10

[104] Behrens JW, Axelsson M, Neuenfeldt S, Seth H. Effects of Hypoxic Exposure during Feeding on SDA and Postprandial Cardiovascular Physiology in the Atlantic Cod, *Gadus morhua*. PLoS ONE 2012;7(9): e46227 10.1371/journal.pone.0046227 23049987 PMC3457987

[105] Houston AH. Blood and Circulation In: Schreck CB, Moyle PB, editors. Methods for Fish Biology. Maryland, American Fisheries Society; 2002 pp. 273–334.

[106] R: A language and environment for statistical computing. R Foundation for Statistical Computing, Vienna, Austria URL https://www.R-project.org/

[107] Schmider E, Ziegler M, Danay E, Beyer L, Bühner M. Is It Really Robust? Reinvestigating the Robustness of ANOVA Against Violations of the Normal Distribution Assumption. Methodology 2010; Vol. 6(4):147–151

