# Supplementary material for: Evaluating dispersal potential of an invasive fish by the use of aerobic scope and osmoregulation capacity
Source: PLoS One. 2017 Apr 19;12(4):e0176038. doi: 10.1371/journal.pone.0176038 (PMC5397051; doi:10.1371/journal.pone.0176038)
Supplement: S1 Table — All individual values of standard metabolic rate (SMR), maximum metabolic rate (MMR) and aerobic scope (AS), in addition to blood plasma osmolality and osmotic gradient, are shown in relation to ambient (treatment) salinity. (PDF) [file pone.0176038.s001.pdf]

Fish:	Fish ID (R=respirometry, O=blood plasma osmolality)
Salinity:	Treatment salinity (PSU)
BM	Body mass in wet weigth (g)
TL	Tail length (mm)
MMR	Maximum metabolic rate (mgO2 kg^{-1} h^{-1})
SMR	Standard metabolic rate (mgO2 kg^{-1} h^{-1})
AS	Aerobic scope (MMR-SMR (mgO2 kg^{-1} h^{-1})
Blood Osm	blood plasma osmolality (mOsm/kg)
Amb Osm	Ambient osmolality (mOsm/kg)
Osm gradient	Osmotic gradient ([Blood osm - Amb osm] (mOsm/kg))

For fish used in respirometry (denoted with R) MMR data from fish R24 and R38 were excluded from the data analysis (see text in Material and Methods for details and resoning). Since AS=MMR-SMR, AS from these two fish was not obtained either.

Fish	Salinity	BM	TL	MMR	SMR	AS
R1	30	45	154	264.20	74.31	189.89
R2	30	64	157	235.80	94.21	141.59
R3	30	57	156	338.10	65.26	272.84
R4	25	57	153	229.80	64.81	164.99
R5	25	50	147	249.80	91.58	158.22
R6	25	50	161	332.30	56.77	275.53
R7	25	44	158	295.20	66.03	229.17
R8	25	37	143	236.90	50.14	186.76
R9	25	37	147	371.28	50.80	320.48
R10	25	34	133	377.14	54.64	322.50
R11	30	47	154	368.16	66.97	301.19
R12	30	38	143	309.94	71.94	238.00
R13	30	42	143	322.50	51.33	271.17
R14	30	43	153	251.20	69.14	182.06
R15	20	60	162	304.84	55.52	249.32
R16	20	73	178	404.78	73.52	331.26
R17	20	66	168	361.20	58.05	303.15
R18	20	43	149	384.12	68.04	316.08
R19	20	49	148	322.09	53.07	269.02
R20	20	50	156	314.12	57.39	256.73
R21	20	49	151	364.00	61.92	302.08
R22	20	43	147	373.43	66.84	306.59
R23	15	78	177	350.20	64.65	285.55
R24	15	54	147	234.70	48.99	185.71
R25	15	74	170	342.20	74.41	267.79
R26	15	48	149	373.50	69.19	304.31
R27	15	43	146	433.70	60.51	373.19
R28	15	41	142	350.30	49.77	300.53
R29	15	74	167	359.60	68.04	291.56
R30	0	39	144	342.90	71.08	271.82
R31	0	52	151	236.70	72.52	164.18
R32	0	63	164	387.00	64.79	322.21
R33	0	46	145	473.96	62.72	411.24
R34	0	41	142	474.12	72.16	401.96
R35	0	76	171	359.10	74.46	284.64
R36	0	50	145	322.70	70.66	252.04
R37	0	56	155	241.40	62.79	178.61
R38	10	72	170	205.40	44.32	161.08
R39	10	59	160	337.00	46.02	290.98
R40	10	43	150	288.80	47.27	241.53
R41	10	53	162	329.24	51.12	278.12
R42	10	51	157	418.10	45.78	372.32
R43	10	39	143	353.70	51.74	301.96
R44	10	36	134	501.40	57.71	443.69
R45	10	70	158	452.50	59.24	393.26
R46	30	25	123	198.54	79.22	119.33
R47	25	50	150	296.70	62.32	234.38
R48	15	35	132	385.60	49.43	336.17

Fish	Salinity	BM	TL	Blood Osm	Amb Osm	Osm gradient
O1	0	45	141	306	4	302
O2	0	37	135	328	4	324
O3	0	82	173	335	4	331
O4	0	127	210	325	4	321
O5	0	82	170	337	4	333
O6	0	53	150	340	4	336
O7	0	63	158	340	4	336
O8	0	53	156	329	4	325
O9	0	70	164	332	4	328
O10	0	45	138	350	4	346
O11	10	50	150	334	263	71
O12	10	57	156	334	263	71
O13	10	67	168	320	263	57
O14	10	68	160	313	263	50
O15	10	54	156	350	263	87
O16	10	38	150	339	263	76
O17	10	58	161	340	263	77
O18	10	88	185	352	263	89
O19	10	57	164	344	263	81
O20	15	50	149	334	422	89
O21	15	100	195	337	422	85
O22	15	74	168	335	422	88
O23	15	47	148	351	422	72
O24	15	74	174	339	422	84
O25	15	42	142	327	422	96
O26	15	86	189	352	422	71
O27	15	80	176	364	422	58
O28	15	47	145	345	422	78
O29	15	52	148	337	422	86
O30	20	77	188	325	601	276
O31	20	59	165	337	601	264
O32	20	48	154	352	601	250
O33	20	66	167	321	601	281
O34	20	65	176	339	601	262
O35	20	47	160	336	601	266
O36	20	99	196	348	601	254
O37	20	47	150	342	601	259
O38	20	51	154	356	601	246
O39	20	42	148	354	601	248
O40	25	48	152	331	747	417
O41	25	51	157	322	747	425
O42	25	49	162	352	747	396
O43	25	50	155	332	747	415
O44	25	52	154	340	747	407
O45	25	77	183	409	747	339
O46	25	40	143	355	747	393
O47	25	77	183	417	747	330
O48	25	42	151	352	747	396
O49	25	48	147	335	747	413
O50	30	88	180	372	844	472
O51	30	62	163	349	844	495
O52	30	45	144	382	844	463
O53	30	54	157	353	844	491
O54	30	62	159	356	844	488
O55	30	45	150	452	844	392
O56	30	40	142	366	844	478
O57	30	41	145	360	844	484
O58	30	44	148	359	844	486

Salinity	Survival (%)
0	93
10	94
15	95
20	89
25	72
30	61
